# Occupational Heat Stress Recommendation Compliance Attenuates AKI Risk Compared with a Work–Rest Ratio–Matched, Positive Control Scenario

**DOI:** 10.34067/KID.0000000000000288

**Published:** 2023-11-01

**Authors:** Hayden W. Hess, Tyler B. Baker, Macie L. Tarr, Roger S. Zoh, Blair D. Johnson, David Hostler, Zachary J. Schlader

**Affiliations:** 1 Department of Kinesiology, School of Public Health-Bloomington, Indiana University, Bloomington, Indiana; 2 Department of Epidemiology and Biostatistics, School of Public Health-Bloomington, Indiana University, Bloomington, Indiana; 3 Department of Exercise and Nutrition Sciences, Center for Research and Education in Special Environments, University at Buffalo, Buffalo, New York

**Keywords:** AKI, renal function, renal injury

## Abstract

- Occupational heat stress recommendations attenuate AKI risk compared with a work–rest ratio–matched positive control scenario.
- Heat-induced AKI risk is strongly related to peak core temperature.
- The peak change in serum creatinine largely paralleled peak changes in urinary [insulin-like growth factor-binding protein 7·tissue inhibitor metalloproteinase 2].

## Introduction

Outdoor workers are regularly exposed to hot environments,^1^ the frequency and intensity of which is predicted to increase because of climate change.^2^ The resulting heat strain, that is, the physiologic effect of heat exposure,^3^ increases the risk of AKI, as supported by findings from workplace,^3^ laboratory-based human subjects,^4,5^ and preclinical^6^ studies. To mitigate the deleterious effects of heat, federal and non-federal entities issue exposure limit recommendations. For example, the US National Institute of Occupational Safety and Health (NIOSH) heat stress recommendations aim to maintain peak core temperature ≤38.0°C in unacclimatized workers.^7^ The recommendations prescribe work–rest ratios on the basis of environmental factors (*e.g.*, wet-bulb globe temperature [WBGT]) and metabolic heat production, and adherence largely ensures that core temperature is ≤38.0°C in unacclimatized adults.^8^ However, it is unknown whether the recommendations protect against heat-induced elevations in AKI risk. We tested the hypothesis that AKI risk is attenuated when adhering to occupational heat stress recommendations.

## Methods

The study was approved by the Institutional Review Board at Indiana University (1902420140), conformed to the Declaration of Helsinki, and was registered at ClinicalTrials.gov (NCT04767347). Before participation, each participant was informed of the procedures and risks before providing informed written consent. The data presented here represent the primary outcome while a portion of these presented were previously published in a study that tested a unique hypothesis.^8^

Twelve healthy adults (6 women; age: 28 years [range, 21–38]; body mass index: 24 kg/m^2^ [range, 18–29]) participated in this study. All participants were free of any chronic disease, were not heat-acclimated, regularly engaged in physical activity, and were nonsmokers. All had normal kidney function (*i.e.*, eGFR >60 ml/min per 1.73 m^2^). Women were not pregnant and self-reported to be normally menstruating. Women were tested at any point during their menstrual cycle.

**Figure 1:**
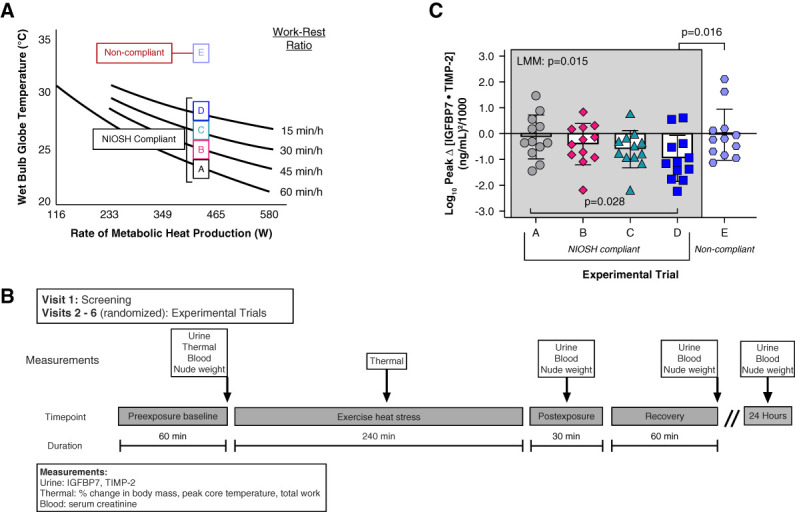
**Study design and primary outcome variable.** (A) Study design and experimental trials on the basis of the NIOSH heat stress recommendations.^7^ Participants walked on a treadmill at NIOSH-compliant work–rest ratios that were prescribed as a function of WBGT and metabolic heat production. Trial E was not NIOSH-compliant, but was used as a positive control with the work–rest ratio matched with trial D. (B) Schematic of the study protocol. In a block-randomized crossover design, participants completed five experimental trials (visits 2–6). (C) Primary outcome variable of log_10_-transformed peak changes (Δ) in urinary [IGFBP7·TIMP-2] data in the NIOSH-compliant (A–D) and non-compliant (E) trials. Data are presented as mean±SD with individual values. Data were analyzed using a one-way repeated-measures LMM for NIOSH-compliant trials (A–D) and using a two-tailed paired *t* test for work–rest ratio–matched compliant versus non-compliant trials (D versus E). Please note that many times, peak Δ urinary [IGFBP7·TIMP-2] was less than one. Log_10_ transformation of integers less than one results in a negative number. This should not be interpreted as a decrease in peak Δ urinary [IGFBP7·TIMP-2] because all raw data were greater than zero. IGFBP7, insulin-like growth factor-binding protein 7; LMM, linear mixed model; NIOSH, National Institute of Occupational Safety and Health; TIMP-2, tissue inhibitor metalloproteinase 2; WBGT, wet-bulb globe temperature.

Participants visited the laboratory on six occasions separated by at least 7 days. The first visit involved screening, and visits 2 through 6 were experimental trials (Figure 1A). The five experimental trials consisted of 4 hours (half a workday) of exposure to WBGTs of 24.1±0.3°C (trial A), 26.6±0.2°C (trial B), 28.4±0.2°C (trial C), 29.7±1.6°C (trial D), and 36.1±0.3°C (trial E). Participants walked on a treadmill evoking an average rate of metabolic heat production (H_prod_) of 430 W, the average H_prod_ for activities commonly completed by outdoor workers.^9^ Average measured H_prod_ was 431±101 W (trial A), 461±106 W (trial B), 462±91 W (trial C), 453±105 W (trial D), and 453±113 W (trial E), which did not differ between trials (*P* = 0.954). NIOSH-compliant^7^ work–rest ratios were prescribed as a function of WBGT and H_prod_ (work:rest min per hour—trial A: 60:0, trial B: 45:15, trial C: 30:30, trial D: 15:45). In trial E, the work–rest ratio was 15:45. Trial E was not NIOSH-compliant, but was used as a positive control with the work–rest ratio matched with trial D. In trial E, the environmental conditions reflected what was previously known as the ceiling limit, defined as the threshold WBGT that NIOSH would stipulate that work would be contraindicated,^7^ thereby rendering trial E non-compliant with the NIOSH recommendations. The experimental trials were completed in a block-randomized order, and participants were blinded to the environmental conditions where possible.

The experimental timeline is presented in Figure 1B. All participants were euhydrated upon arrival, defined as a urine specific gravity ≤1.020.^10^ After urine collection, participants drank 376±61 ml (0.5% body mass) of cool tap water. They then rested supine for approximately 60 minutes in an approximately 22°C environment. A blood sample was then obtained; they voided their bladder, were weighed nude, and then donned long pants and shirt, a short-sleeve cotton T-shirt, and athletic shoes. Participants then entered the environmental chamber and the four-hour exposure commenced. Compliant with the NIOSH guidelines,^7^ they were provided 237 ml of a cool (9.2±4.0°C) preferred flavor sport drink (Gatorade) every 15 minutes and were permitted to drink *ad libitum*. During rest periods, participants sat on a mesh chair. After the exposure, they were weighed nude, voided their bladder, and then rested supine for 20 minutes before a venous blood sample was collected. They then rested for approximately 60 minutes before measurements were repeated. They returned to the laboratory approximately 24 hours after the start of the experimental trial for a final urine and blood sample.

Core temperature was measured using a telemetry pill (HQ Inc.) swallowed approximately 6–8 hours before each experimental trial (*n*=8) or a rectal temperature probe inserted approximately 10 cm beyond the sphincter (*n*=4) for participants contraindicated for taking the telemetry pill. Serum creatinine (sCr) was measured using human creatinine ELISA kits (Eagle Bioscience, Inc.). Insulin-like growth factor-binding protein 7 (IGFBP7) and tissue inhibitor metalloproteinase 2 (TIMP-2) were measured in urine using separate commercially available (RayBiotech Life) human IGFBP7 and TIMP-2 ELISA kits. Consistent with the US Food and Drug Administration–approved indication in clinical settings, the product of urinary IGFBP7 and TIMP-2 ([IGFBP7·TIMP-2]) provided an index of AKI risk.^11^

Previously published data^11^ revealed a Cohen d_z_ effect size of 0.75 for peak urinary [IGFBP7·TIMP-2] evoked during physical work in the heat with and without body cooling (mean±SD difference: −1.4±1.9 [ng/ml]^2^/1000). Before study commencement, a power analysis using this effect size and standard parameters of 1−*β*=0.80 and *α*=0.05 and a moderate correlation between repeated measures (*r*=0.5) using G*Power software (v. 3.1.9.4) revealed that we needed 12 participants to complete this study.

Core temperature is presented as the highest recorded value.^7^ Urinary [IGFBP7·TIMP-2] is presented as the peak change (Δ) from pre-exposure, which is consistent with the US Food and Drug Administration–approved indication where time course and correction to urine concentration are not considered.^12^ The peak change in urinary IGFBP7 and TIMP-2 were independently normalized to urine osmolality to account for urine concentration. Percent changes in body mass from pre- to postexposure provided an index of changes in total body water.^10^ The peak change in sCr provided an index of changes in kidney function.^13^ Total work during each trial was calculated from treadmill speed and grade, body weight, walking time, and acceleration due to gravity. Only two participants were able to complete the entire four-hour period in trial E (exhaustion: *n*=7; core temperature of 39.5°C: *n*=3).

Four unique statistical analyses were undertaken. First, we examined the dependent variables across the NIOSH-compliant scenarios using repeated-measures one-way linear mixed models, and when a significant effect was identified, multiple comparisons were performed using the Tukey test. Second, we compared dependent variables between the NIOSH-compliant (trial D) and non-compliant (trial E) conditions using two-tailed paired *t* tests. This permitted direct assessment of whether compliance with NIOSH recommendations modified AKI risk when work–rest ratios were matched. Third, we examined relations between peak Δ urinary [IGFBP7·TIMP-2] and sCr and these variables as a function of total work and peak core temperature using Pearson correlations. Finally, we examined the dependent variables when peak core temperature was ≤38.0°C versus >38.0°C using two-tailed unpaired *t* tests assuming unequal variance between groups. After visual inspection of predicted and actual residuals, we identified that peak Δ urinary [IGFBP7·TIMP-2], IGFBP7, and TIMP-2 were not normally distributed. Therefore, all statistical analyses of these variables were completed after log_10_ transformation, which resulted in the data being normally distributed. Many times, peak Δ urinary [IGFBP7·TIMP-2] was less than one. Log_10_ transformation of integers less than one result in a negative number. This should not be interpreted as a decrease in peak Δ urinary [IGFBP7·TIMP-2] because all raw data were greater than zero. Data were analyzed with GraphPad Prism software (version 8). Data are presented as individual values and/or absolute mean±SD. A priori statistical significance was set at *P* ≤ 0.05.

## Results

**Figure 2:**
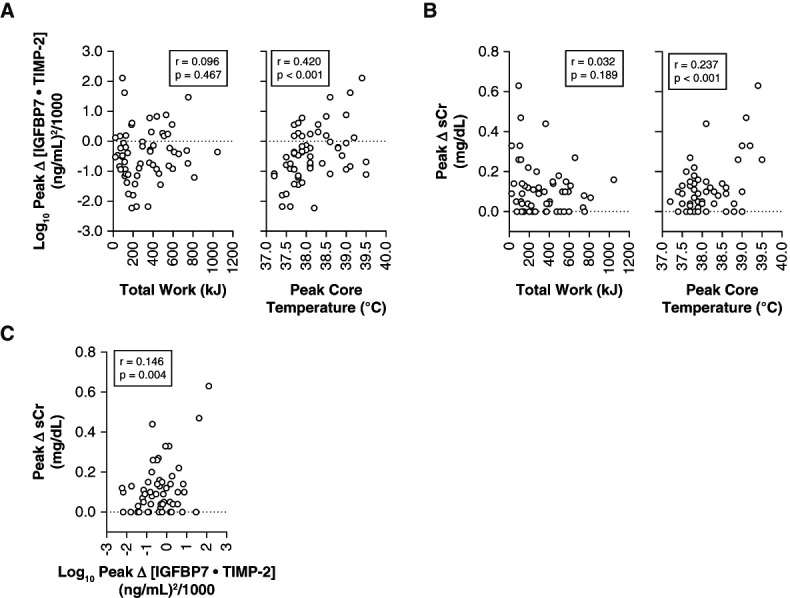
**Correlation analyses between AKI risk and potential modulators.** (A) Pearson correlation between log_10_-transformed peak Δ urinary [IGFBP7·TIMP-2] and total work, peak core temperature. (B) Pearson correlation between peak Δ in sCr and total work, peak core temperature. (C) Pearson correlation between peak Δ in sCr and peak Δ urinary [IGFBP7·TIMP-2]. Please note that many times, peak Δ urinary [IGFBP7·TIMP-2] was less than one. Log_10_ transformation of integers less than one results in a negative number. This should not be interpreted as a decrease in peak Δ urinary [IGFBP7·TIMP-2] because all raw data were greater than zero. sCr, serum creatinine.

Log_10_ peak Δ urinary [IGFBP7·TIMP-2] across the NIOSH-compliant and non-compliant trials is presented in Figure 1C. The physiologic responses during these trials are presented in Table 1. Pearson correlations are presented in Figure 2. Table 2 presents AKI risk data in those observations with peak core temperature ≤38.0°C versus >38.0°C.

**Table 1:** Physiologic responses during the experimental trials

Experimental Trial	NIOSH-Compliant				Non-Compliant	*P* Values	
	A	B	C	D	E	LMM (A–D)	Paired *t* Test (D versus E)
Peak core temperature, °C	38.1±0.4	37.9±0.4	37.9±0.3	37.7±0.4	39.1±0.3	0.065	<0.001
Δ body weight, %	−0.8±0.7	−0.7±0.4	−0.5±0.3	−0.3±0.6	−0.7±1.0	0.131	0.185
Total work, kJ	617±234	450±157	299±104	151±52	97±34	<0.001^a^	<0.001
Log_10_ peak Δ IGFBP7, ng/mOsm	5.16±1.28	5.23±0.51	5.23±0.65	4.95±0.37	5.87±1.03	0.673	0.014
Log_10_ peak Δ TIMP-2, ng/mOsm	1.88±1.24	1.84±0.87	1.56±1.34	1.14±1.27	2.77±1.12	0.241	0.002
Peak Δ sCr, mg/dl	0.08±0.07	0.12±0.13	0.07±0.07	0.06±0.07	0.25±0.21	0.326	0.036

Data are presented as mean±SD. Δ Indicates change from pre-exposure. *N*=12 except for peak Δ serum creatinine, where *n*=11 in trials A and B and *n*=10 in trial E. Data were analyzed using a one-way linear mixed model for National Institute of Occupational Safety and Health–compliant trials (A-D) and using a two-tailed paired *t* test for work:rest ratio–matched compliant versus non-compliant trials (D versus E). Data for peak Δ insulin-like growth factor-binding protein 7 and tissue inhibitor metalloproteinase 2 were normalized to urine osmolality (ng/mOsm) to account for differences in urine concentration between trials and were analyzed after log_10_ transformation. IGFBP7, insulin-like growth factor-binding protein 7; LMM, linear mixed model; NIOSH, National Institute of Occupational Safety and Health; sCr, serum creatinine; TIMP-2, tissue inhibitor metalloproteinase 2.

a Indicates all National Institute of Occupational Safety and Health–compliant trials (*i.e.*, A–D) different from each other (all *P* < 0.001).

**Table 2:** AKI risk in those observations above versus below a core temperature of 38.0°C

Group	≤38.0°C	>38.0°C	*P* Values
All trials, *n* (#)	33	27	—
Compliant/Non-compliant trials, *n* (#)	33/0	15/12	—
Peak core temperature, °C	37.7±0.2	38.7±0.5	<0.001
Total work, kJ	356±237	282±229	0.223
Log_10_ peak Δ urinary (IGFBP7·TIMP-2), (ng/ml)^2^/1000	−0.58±0.76	−0.13±0.95	0.019
Peak Δ sCr, mg/dl	0.07±0.08 (*n*=32)	0.16±0.17 (*n*=24)	0.021

Data are presented as mean±SD. Δ Indicates change from pre-exposure. Data were analyzed using a two-tailed unpaired paired *t* test assuming unequal variances between groups. Data for peak Δ urinary [IGFBP7·TIMP-2] were analyzed after log_10_ transformation. Note that log_10_ transformation of integers less than one result in a negative number. This should not be interpreted as a decrease in peak Δ urinary [IGFBP7·TIMP-2] because all raw data were greater than zero. IGFBP7, insulin-like growth factor-binding protein 7; sCr, serum creatinine; TIMP-2, tissue inhibitor metalloproteinase 2.

## Discussion

Adherence to the NIOSH heat stress recommendations attenuated AKI risk compared with a work–rest ratio–matched, positive control scenario. Within the NIOSH-compliant scenarios, peak Δ urinary [IGFBP7·TIMP-2] was highest in the coolest condition in which a work–rest ratio is not prescribed. Changes in these urinary biomarkers are not likely due to differences in urine concentration or changes in total body water. Rather, AKI risk was strongly related to peak core temperature and not total work, both of which differed between the trials. The importance of peak core temperature on AKI risk is further supported by the finding that peak Δ urinary [IGFBP7·TIMP-2] was higher in observations in which peak core temperature exceeded 38.0°C, despite no differences in total work between these groups. This finding supports the NIOSH recommendation to ensure peak core temperature does not exceed 38.0°C in unacclimated workers.^7^ Peak Δ sCr was correlated with the peak Δ urinary [IGFBP7·TIMP-2], and these variables largely changed in parallel, supporting the use of changes in sCr as a marker of AKI risk in both laboratory and field settings. Future work should examine the effect of heat acclimatization on heat-induced AKI risk.

## Data Availability

All data are included in the manuscript and/or supporting information. Previously published data were used for this study. Hess HW, Tarr ML, Baker TB, Hostler D, Schlader ZJ: Ad libitum drinking prevents dehydration during physical work in the heat when adhering to occupational heat stress recommendations. Temperature, 2022.
